# Comparative Evaluation of Er: YAG Laser, Diode Laser, and Novamin Technology for Dentinal Tubule Occlusion: An In-Vitro Scanning Electron Microscope (SEM) and Energy Dispersive X-Ray Analysis (EDX) Study

**DOI:** 10.7759/cureus.58806

**Published:** 2024-04-23

**Authors:** Vivek Srivastava, Shreya Haldar, Vipul Srivastava, Ajita Meenawat, Yasir Shahab Khan, Elizabeth Huidrom

**Affiliations:** 1 Department of Periodontology, Sardar Patel Post Graduate Institute of Dental and Medical Sciences, Lucknow, IND; 2 Department of Conservative Dentistry and Endodontics, 32 Pearls Dental Clinic, Lucknow, IND

**Keywords:** energy dispersive x-ray analysis, scanning electron microscope, lasers, dentinal tubule occlusion, dentinal hypersensitivity

## Abstract

Background: Dentinal hypersensitivity is a brief and painful oral condition that is characterized by an abrupt shooting sensation. Stimulation occurs when hot, cold, sweet, or sour food comes into contact with exposed dentinal tubules. The present study used a scanning electron microscope (SEM) and energy dispersive X-ray analysis (EDX) to investigate the efficacy of Er: YAG, 810 nm diode LASER, and NovaMin Technology in obstructing dentinal tubules.

Material and methods: We extracted the outer layers of 30 human teeth to expose the tubules and then treated the surfaces with 17% ethylenediaminetetraacetic acid (EDTA) to create an etched effect. Three cohorts were created from the portions. Group A was subjected to the application of Erbium:Yttrium-Aluminum-Garnet (Er: YAG) laser with a power output of 2W in the non-contact mode for 1 minute. Group B was subjected to the application of an 810nm diode laser with a power output of 1W in continuous mode for 30 seconds. Group C was subjected to the application of NovaMin paste, which contains a 927 ppm fluoride content. Following the therapy, occluded dentinal tubules were analyzed using scanning electron microscopy (SEM) and energy-dispersive X-ray spectroscopy (EDX) for both quantitative and qualitative examination. The data analysis was conducted using a one-way analysis of variance (ANOVA) and Tukey's test, with a significance threshold of 0.05.

Results: The average percentages of complete blockage of dentinal tubules in Groups A, B, and C were evaluated using the number of entirely unobstructed dentinal tubules at magnifications of 2000X (F = 3.05, p = 0.064), 5000X (F = 5.33, p = 0.011), and 10000X (F = 8.63, p = 0.001). The count of partially open dentinal tubules seen at magnifications of 2000X, 5000X, and 10000X was F = 10.15 (P < 0.001), F = 5.97 (p = 0.007), and F = 2.12 (p = 0.140) accordingly.

Conclusion: NovaMin technology has demonstrated more effectiveness in blocking dentinal tubules compared to 810nm diodes and Er: YAG lasers.

## Introduction

The term dentinal hypersensitivity (DH) refers to pain that originates from exposed dentine and is caused by many stimuli, such as thermal, evaporative, tactile, osmotic, or chemical, and cannot be associated with any other dental defect or condition [1]. 4-74% of people experience this type of pain, which is characterized by its fleeting, shooting nature and quick response. The pain is transient and characterized by its brief and shooting nature, with an instantaneous response felt [2-4]. DH has been caused by the frequent consumption of food and acidic drinks and the population of eating disorders, such as anorexia and bulimia. Various theories of hypersensitivity, like the transducer theory, the modulation theory, the “gate” control and vibration theory, and the hydrodynamic theory, have been put forward, out of which the hydrodynamic theory, given by Brannstrom and his co-workers, was solidified as the primary understanding of DH [1,5,6].

Desensitizing toothpastes, dentifrices with potassium salts, and topical desensitizing agents were some of the initial therapy modalities. An alternative treatment plan, such as laser treatment, adhesive resin bonding, primers, sealants, varnishes, conventional glass ionomer cement, and resin-reinforced glass ionomer, may be provided by additional professional care. These treatments have demonstrated differing degrees of effectiveness in treating dentine hypersensitivity, with some studies recommending their use and others indicating that they are ineffective [7-9]. Erbium: Yttrium-Aluminum-Garnet (Er: YAG) laser therapy is successful, but more recently, the diode laser demonstrated its potential for treating DH by successfully occluding the exposed dentinal tubules [10,11]. Nevertheless, the desensitizing agent NovaMin creates crystals resembling hydroxyapatite that block exposed dentinal tubules in teeth [12].

The major aim and objective of the present study was to see the efficacy of Er: YAG LASER, Diode LASER, and NovaMin and to evaluate dentinal tubule occlusion (both partially and completely occluded tubules) under a scanning electron microscope (SEM) and the presence of inorganic components under Energy Dispersive X-ray Analysis (EDX), providing the chemical mapping of any surface deposits that can be useful in characterizing agents used to occlude dentinal tubules for the treatment of DH [13,14].

## Materials and methods

**Figure 1:**
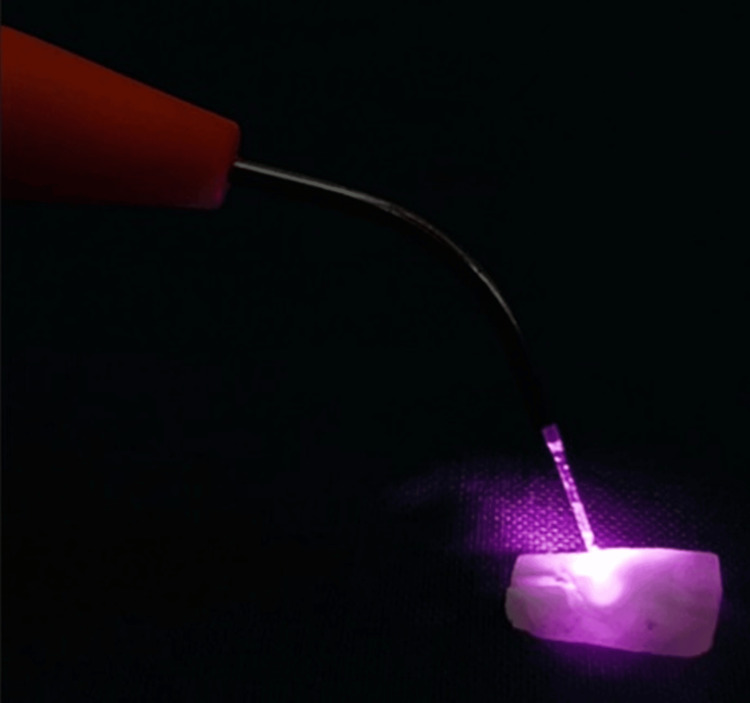
Group B specimen is treated with Diode LASER

Thirty human-extracted teeth were included with criteria inclusive of intact crowns and root surfaces, non-restorative, and non-carious teeth with no enamel anomalies. It should be free of resorbed and ankylosed roots with signs of fracture and discoloration. Extracted teeth were divided into three treatment modalities, Er: YAG LASER* (*FOTONA 80 SLOVENIA) at 2790nm (Group A), diode LASER† (†Picasso AMD lasers, USA) at 810nm (Group B), and NovaMin paste‡ (‡SENSODYNE REPAIR & PROTECT®) (Group C) (Figure 1). 

The human-extracted teeth were cleaned, and scaling and root planing were done with ultrasonic scalers and Gracey curettes. The crowns were decorated, and the buccal/facial surface was sliced using carborundum disc bur, and the surface was polished with Arkansas stone, maintaining a thickness of 2mm. Etching was done with 17% EDTA for 40 minutes and washed in distilled water for 2 minutes to completely open dentinal tubules [15].

**Figure 2:**
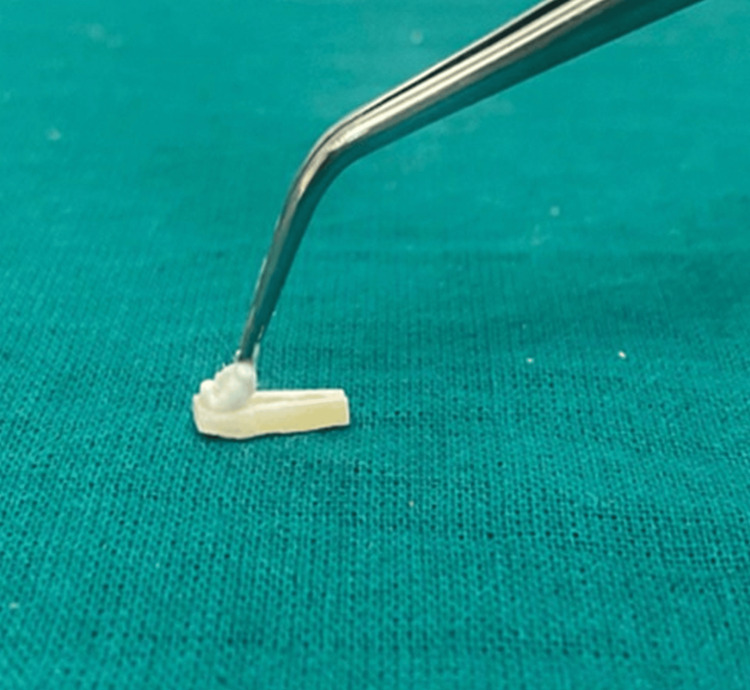
Group C specimen is treated with NovaMin paste

In Group A, 10 buccal/facial surfaces were treated using Er: YAG LASER* (2790nm) at 2W in the non-contact mode for 30sec, spot size 1cm^2^, and an energy density of 60J/cm^2^. In Group B, 10 buccal/facial surfaces were treated with diode LASER† (810nm) at 1W in continuous mode for 15sec, spot size 1cm^2^ with an energy density of 15J/ cm^2^, and in Group C, the 10 buccal/facial surfaces were treated using NovaMin paste‡ (927ppm fluoride content) in a cotton pellet for 3 min. All the specimens from each group were kept in Hank’s Balanced Salt Solution (HBSS) for one month (Figure 2).

Preparation of samples for SEM analysis

**Figure 3:**
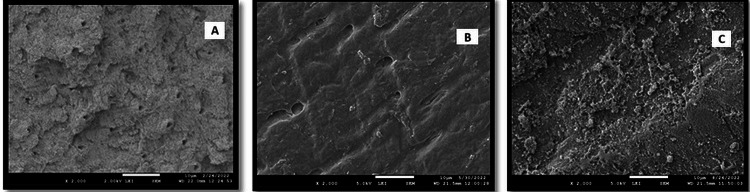
SEM analysis of Group A, Group B, and Group C at 2000X

For scanning electron microscope analysis, the specimens were placed in 2.5% glutaraldehyde in 0.1M HBSS for a minimum of 24 hours [3], followed by washing and dehydration through a graded alcohol series (25%, 50%, 70%, 90%, and 100%) for 10 minutes each, after which specimens will be mounted on SEM stubs of 1 cm2. These mounted specimens will be air dried for 48 hours and sputter coated with 30-40 nm of gold using an ion sputtering device§ (§ JEOL, JEC-3000FC). These specimens were examined under SEMk (kJEOL 7610F) by operating at an accelerating voltage of 40 kV. The parameters chosen were tubular diameter and occlusion. Under SEM, each specimen was scanned at variable magnifications (X2, X5, and X10) to identify the dentinal tubules, both partially and completely occluded, and was obtained in a longitudinal section [16] (Figure 3).

Energy dispersive X-ray (EDX) analysis

In energy dispersive X-ray (EDX), all of the specimens will be examined at 20 kV with a spot size of 5nm and a counting time of 300 s to qualitatively determine the presence of chemical elements such as carbon (C), calcium (Ca), phosphorus (P), sodium (Na), potassium (K), and oxygen (O).

Statistical analysis

One-factor analysis of variance (ANOVA) was used to compare the groups, and Tukey's HSD (honestly significant difference) post hoc test was used to determine the significance of the mean difference between the groups after Shapiro-Wilk's test and Levene's test had determined the homogeneity of variance between the groups.

Ethical consideration

The ethical approval for the study was obtained from the Sardar Patel Post Graduate Institute of Dental and Medical Sciences with institutional review board number (IRB) PERIO/05/752021/IEC.

## Results

Scanning electron microscopic examination provided photomicrographs that were obtained at X2000, X5000, and X10000 magnifications at 40 kV voltage. The mean number of fully open dentinal tubules decreases with an increase in magnification (10000X < 5000X < 2000X). ANOVA showed a similar mean number of fully open dentinal tubules among the groups (F = 3.05, P = 0.064) at magnification 2000X. However, fully open dentinal tubules at both 5000X (F = 5.33, P = 0.011) and 10000X (F = 8.63, P = 0.001) magnifications differed significantly among the groups (Table 1).

**Table 1:** Number of full-open dentinal tubules of three groups at three different magnifications The number of dentinal tubules full open in three groups at each of the three magnifications was summarized in Mean ± SE and compared by ANOVA (F value). A p-value was considered significant if it was <0.05.

Group	n	Magnification 2X	Magnification 5X	Magnification 10X
Group A	10	3.60 ± 0.58	2.20 ± 0.39	1.70 ± 0.30
Group B	10	2.50 ± 0.76	1.00 ± 0.30	0.80 ± 0.13
Group C	10	1.40 ± 0.52	0.90 ± 0.23	0.50 ± 0.17
P-value		0.064	0.011	0.001

On inter-group comparison, the Tukey test showed a similar (P > 0.05) mean number of fully open dentinal tubules between the three groups at magnification 2000X (Table 2).

**Table 2:** For each magnification, comparison (p-value) of the difference in mean number of dentinal tubules full open between groups by the Tukey test diff: difference, CI: confidence interval, q value: Tukey test value A p-value was considered significant if it was <0.05.

Magnification	Comparison	Mean diff.	q value	p-value	95% CI of diff.
2X	Group A vs. Group B	1.10	1.75	0.09	-1.112 to 3.312
	Group A vs. Group C	2.20	3.49	0.21	-0.012 to 4.412
	Group B vs. Group C	1.10	1.75	0.08	-1.112 to 3.312
5X	Group A vs. Group B	1.20	3.83	0.02	0.101 to 2.299
	Group A vs. Group C	1.30	4.15	0.01	0.201 to 2.399
	Group B vs. Group C	0.10	0.32	0.07	-0.999 to 1.199
10X	Group A vs. Group B	0.90	4.23	0.01	0.154 to 1.646
	Group A vs. Group C	1.20	5.65	0.001	0.454 to 1.946
	Group B vs. Group C	0.30	1.41	0.11	-0.446 to 1.046

However, on comparison at both 5000X and 10 X magnifications, the mean number of fully open dentinal tubules lowered significantly (p < 0.05 or p < 0.01) in both Group B and Group C as compared to Group A but did not show statistically significant differences (p > 0.05) between Group B and Group C.

ANOVA showed a significantly different mean number of partially open dentinal tubules among the groups at both 2000X (F = 10.15, p < 0.001) and 5000X (F = 5.97, p = 0.007) magnifications. At magnification 10000X, it was found to be similar among the other groups (F = 2.12, p = 0.140). The number of partially open dentinal tubules in three groups at each of the three magnifications was summarized in Mean ± SE and compared by ANOVA (F value) (Table 3). 

**Table 3:** Number of dentinal tubules that are partially open in three groups at three different magnifications The number of dentinal tubules that were partially open in three groups at each of the three magnifications was summarized in mean ± SE and compared by ANOVA (F value). A p-value was considered significant if it was <0.05.

Group	n	Magnification 2X	Magnification 5X	Magnification 10X
Group A	10	7.10 ± 1.30	3.10 ± 0.57	1.40 ± 0.45
Group B	10	3.40 ± 0.72	1.10 ± 0.28	0.60 ± 0.22
Group C	10	1.50 ± 0.43	1.20 ± 0.49	0.60 ± 0.22
p-value		0.001	0.007	0.140

On inter-group comparison, the Tukey test showed significantly (p < 0.05 or p < 0.001) different and lower mean numbers of partially open dentinal tubules in both Group B and Group C as compared to Group A at both 2000X and 5000X magnifications. It did not differ (p > 0.05) between Group B and Group C (Table 4), and at magnification 10000X, it did not have a significant difference (p > 0.05) among all three groups.

**Table 4:** For each magnification, comparison (p-value) of the difference in mean number of dentinal tubules partial open between groups by the Tukey test diff: difference, CI: confidence interval, q value: Tukey test value. A p-value was considered significant if it was <0.05.

Magnification	Comparison	Mean diff.	q value	p-value	95% CI of diff.
2X	Group A vs. Group B	3.70	4.14	0.03	0.563 to 6.837
	Group A vs. Group C	5.60	6.26	0.001	2.463 to 8.737
	Group B vs. Group C	1.90	2.13	0.07	-1.237 to 5.037
5X	Group A vs. Group B	2.00	4.34	0.01	0.382 to 3.618
	Group A vs. Group C	1.90	4.12	0.04	0.282 to 3.518
	Group B vs. Group C	-0.10	0.22	0.08	-1.718 to 1.518
10X	Group A vs. Group B	0.80	2.52	0.09	-0.314 to 1.914
	Group A vs. Group C	0.80	2.52	0.08	-0.314 to 1.914
	Group B vs. Group C	0.00	0.00	0.06	-1.114 to 1.114

EDX is used to analyze the presence of chemical elements like carbon (C), calcium (Ca), phosphorus (P), sodium (Na), potassium (K), and oxygen (O). In all three groups, the weight (%) of element OK was the maximum, and NaK was the minimum (Table 5).

**Table 5:** Distribution of presence of element weight (%) among three groups CK: carbon potassium, OK:  oxygen potassium, PK: phosphorus potassium, CaK: calcium potassium, and NaK: sodium potassium.

Element	Group A	Group B	Group C
CK	13.89	15.76	35.81
OK	45.00	52.17	43.74
PK	14.33	12.40	8.10
CaK	23.46	19.67	12.36
NaK	3.32	0.00	0.00

## Discussion

The present study was designed to evaluate the efficacy of dental LASERs and the bioactive properties of desensitizing toothpaste with the ability to occlude the dentinal tubules. The diagnosis of DH is crucial for confirming its accuracy and ensuring successful treatment while also eliminating other potential causes of pain. Treatments include removing etiologic factors causing DH based on the severity of periodontium health, poor oral hygiene, erosive agents, occlusion correction, and improper brushing techniques [17-19]. The present study determined the efficacy of dental LASERs such as Diode LASER (810nm), Er: YAG LASER (2940nm), and NovaMin technology on the odontoblastic process in the dentinal tubules and was done at fully open and partially open dentinal tubules at three different magnifications.

At 2000X (p = 0.064) magnification, the number of fully open dentinal tubules in the Er: YAG LASER-treated group was found to be higher, hence less ineffective as compared to the Diode LASER and NovaMin-treated groups. A scanning electron microscope (SEM) showed a highly charred and melted surface of the dentinal tubules in the Er: YAG LASER group, which did not help in occluding dentinal tubules, and a similar result was concluded in a study conducted by Belal and Yassin in 2014 [20]. In the group treated by Diode LASER, fully open dentinal tubules at 2000X magnifications showed a better response to dentinal tubule occlusion when compared to Er: YAG LASER. However, the diode group showed even fewer occluded dentinal tubules as compared to NovaMin. Fully open dentinal tubules in the group treated with NovaMin at 2000X magnification showed increased tubule occlusion compared to the Er: YAG and Diode LASER groups.

At 5000X magnification, the Er: YAG-treated group showed a maximum number of fully open dentinal tubules, showing a poor desensitizing effect on fully open dentinal tubules. It was reported that Er: YAG LASER application caused minimal thermal damage to dentin when the energy parameters were 60 mJ/pulse and 30 Hz, which were consistent with the study already conducted by Cakar et al., 2008 [21], Ipci et al., 2009 [22], Tunar et al., 2014 [23]. The mean number of fully open dentinal tubules in the group treated with diode LASER at magnification 5000X was maximum as compared to the group treated with NovaMin, proving the maximum number of dentinal tubules occlusion and the minimum number of occluding dentinal tubules in the Er: YAG-treated group. While comparing the fully open dentinal tubules of the NovaMin technology group at 5000X magnification, it showed significant occlusion of dentinal tubules.

At 10000X (p = 0.001), fully open dentinal tubules showed decreased dentinal tubules because of increased magnification. However, fully open dentinal tubules were last seen in the NovaMin-treated group. At 2000X (p < 0.001) magnification, Er: YAG showed a maximum number of partially open dentinal tubules, indicating a less effective response to desensitization. The diode LASER-treated group demonstrated a lesser number of partially open dentinal tubules than the Er: YAG-treated group, suggesting an improved outcome. A greater number of partially occluded dentinal tubules were seen when the NovaMin-treated group was compared to the Er: YAG-treated group, showing the effectiveness of NovaMin technology as a desensitizing agent.

The Er: YAG-treated group had the most partially open dentinal tubules at 5000X (p = 0.007) magnification compared to the diode lasers and NovaMin-treated groups, while the NovaMin group exhibited negligible improvement. At a magnification of 5000X, the diode laser even produced superior results. Due to its low absorption in hard tissues, an in-vitro scanning electron microscopy study revealed that the specimens treated with an 810nm diode laser exhibited the least morphologic changes [24-28]. Hydroxyapatite crystals have a low absorption of light at 810 nm, which permits laser energy to be transmitted, scattered, and propagated through the dentin. This causes the dentin to heat up and melt, obstructing the dentinal tubules [27]. Diode lasers performed better, according to George VT et al. (2016) [29], in terms of treatment longevity and cost-benefit ratios. Diode LASER (810 nm) provided a decrease in cervical dentine hypersensitivity. Gojkov-Vukelic M et al., 2016 [30], Asnaashari M et al., 2013 [31], and Suri I et al., 2016 [32] concluded in their research that diode LASER with wavelengths between 780 and 810 nm has an effect on nerve endings that eliminates cervical dentinal sensitivity.

The partially open dentinal tubules at magnification 5000X were also observed in the Er: YAG group. Samples irradiated by a high-power Er: YAG laser can induce protein denaturation of the organic matrices and a reduction in crystal surface area. When using the Er: YAG, the surface of the tubules had an open tubular appearance, although a deeper closure with crystallization was found in the cross-sectional image. According to Ishikawa et al. (2003), the accumulation of organic elements or insoluble salts was observed by SEM at the site of blockade and reduction of dentinal tubules by Er: YAG laser desensitization [25]. However, emphasis should be placed on the dose-related response with the high-power laser application and the higher wear of dentine tissues.

NovaMin technology showed better occlusion of dentinal tubules against diode laser and poorer results at occluding dentinal tubules as compared to the Er: YAG-treated group, proving it less significant. The aforementioned outcomes can be linked to dentine's remineralization characteristics and increased hardness as a result of fluoride's synergistic action. Hydrogen ions are exchanged for sodium in NovaMin. Moreover, phosphate and calcium are released into the environment. The calcium and phosphate ions that are released create a Ca-P layer on the tooth's surface, which crystallizes into hydroxycarbonate apatite when the pH briefly rises. In accordance with our study, Shivaprasad BM et al. (2016) concluded that NovaMin has the added advantage of chair-side application [26]. Burwell et al. (2010) [33] showed that one-time brushing with NovaMin-based dentifrice significantly decreased the visible open tubules adhered to the exposed dentin surface layer. This layer is resistant to acid challenges and is mechanically strong due to the continuous release of calcium. However, the present study results were similar when compared with the group treated with diode LASER.

The primary elements present in the treated samples were analyzed using EDX to look for differences in their distribution. Calcium (Ca), potassium (K), phosphorus (P), oxygen (O), and carbon (C) were the main elements found. Table 5 provides a summary of the Ca, K, P, O, and C weight percentages found in the samples. According to EDX analysis, NaK% presence was negligible in the diode laser and Novamin-treated groups. OK% elements were discovered to be more prevalent in Diode LASER irradiation, and they were present in Er: YAG LASER. The crystalline tooth structure suddenly expanded as a result of the Er: YAG LASER's strong interaction with dentin's structures bound to water. By damaging the peritubular dentin, it causes the dentinal tubules to break down irregularly [34]. However, EDX involves only the presence of entrapped molecular elements and is unable to show molecular characteristics.

Study limitations are that the relatively small sample size of 30 healthy human teeth may not fully capture the variability present in the general population, potentially limiting the generalizability of the findings. Moreover, the short-term evaluation immediately after treatment may not reflect the long-term durability and efficacy of the interventions, necessitating further longitudinal studies. Being a single-center study, the results might not be broadly applicable to diverse clinical settings and populations, warranting validation through multicenter trials. Addressing these limitations in future research endeavors would provide a more comprehensive understanding of the utility of Er: YAG LASER, 810nm Diode LASER, and NovaMin technology in managing dentinal hypersensitivity effectively.

## Conclusions

The results of this study indicate that, in the long run, sustained alleviation for acute diarrheal illness (DH) has been demonstrated by SEM analysis, with NovaMin toothpaste emerging as the best option. Therefore, using a diode laser (810 nm) also produced superior results, and this should be taken into account while creating a treatment plan for DH that offers instant relief. Compared to higher-powered lasers, low-power lasers have the advantage of having a lesser potential to cause heat damage to tooth tissue. Future research would greatly benefit from EDX chemical mapping of these crystals to verify the components present in the cross-sectional SEM pictures.
